# Preoperative and early postoperative seizures in patients with glioblastoma—two sides of the same coin?

**DOI:** 10.1093/noajnl/vdaa158

**Published:** 2020-11-18

**Authors:** Yahya Ahmadipour, Laurèl Rauschenbach, Alejandro Santos, Marvin Darkwah Oppong, Lazaros Lazaridis, Carlos M Quesada, Andreas Junker, Daniela Pierscianek, Philipp Dammann, Karsten H Wrede, Björn Scheffler, Martin Glas, Martin Stuschke, Ulrich Sure, Ramazan Jabbarli

**Affiliations:** 1 Department of Neurosurgery and Spine Surgery, University Hospital Essen, Essen, Germany; 2 German Cancer Consortium, Partner Site University Hospital Essen, Essen, Germany; 3 DKFZ-Division Translational Neurooncology at the WTZ, German Cancer Research Center (DKFZ) Heidelberg and German Cancer Consortium (DKTK) Partner Site University Hospital Essen, Essen, Germany; 4 Division of Clinical Neurooncology, Department of Neurology, University Hospital Essen, Essen, Germany; 5 Department for Neurology, University Hospital Essen, Essen, Germany; 6 Department of Neuropathology, University Hospital Essen, Essen, Germany; 7 Department of Radiotherapy, University Hospital Essen, Essen, Germany

**Keywords:** epilepsy, glioblastoma, predictor, survival

## Abstract

**Background:**

Symptomatic epilepsy is a common symptom of glioblastoma, which may occur in different stages of disease. There are discrepant reports on association between early seizures and glioblastoma survival, even less is known about the background of these seizures. We aimed at analyzing the risk factors and clinical impact of perioperative seizures in glioblastoma.

**Methods:**

All consecutive cases with de-novo glioblastoma treated at our institution between 01/2006 and 12/2018 were eligible for this study. Perioperative seizures were stratified into seizures at onset (SAO) and early postoperative seizures (EPS, ≤21days after surgery). Associations between patients characteristics and overall survival (OS) with SAO and EPS were addressed.

**Results:**

In the final cohort (*n* = 867), SAO and EPS occurred in 236 (27.2%) and 67 (7.7%) patients, respectively. SAO were independently predicted by younger age (*P* = .009), higher KPS score (*P* = .002), tumor location (parietal lobe, *P* = .001), GFAP expression (≥35%, *P* = .045), and serum chloride at admission (>102 mmol/L, *P* = .004). In turn, EPS were independently associated with tumor location (frontal or temporal lobe, *P* = .013) and pathologic laboratory values at admission (hemoglobin < 12 g/dL, [*P* = .044], CRP > 1.0 mg/dL [*P* = 0.036], and GGT > 55 U/L [*P* = 0.025]). Finally, SAO were associated with gross-total resection (*P* = .006) and longer OS (*P* = .030), whereas EPS were related to incomplete resection (*P* = .005) and poorer OS (*P* = .009).

**Conclusions:**

In glioblastoma patients, SAO and EPS seem to have quite different triggers and contrary impact on treatment success and OS. The clinical characteristics of SAO and EPS patients might contribute to the observed survival differences.

**Key Points**

- Perioperative epilepsy independently predicts glioblastoma survival.
- Seizures at onset are associated with longer survival after glioblastoma surgery.
- In contrast, early postoperative seizures are related to poor outcome.

**Importance of the Study**

To date, there is a large discrepancy regarding the rate, risk factors, and predictive value of perioperative epileptic seizures in patients with glioblastoma. In this large consecutive glioblastoma cohort, we evaluated over 100 patient/tumor-specific variables with regard to the association with seizures at onset (SAO) and early postoperative seizures (EPS). SAO were independently associated with younger age, better preoperative clinical performance, higher GFAP expression, and higher serum chloride levels. In addition, SAO were related to more radical extent of resection and longer overall survival. In contrast, EPS were strongly associated with presence of systemic disorders like anemia, infection, and liver dysfunction as well as incomplete tumor resection and poorer overall survival. Our results encourage further analysis of the effect of perioperative seizures on glioblastoma survival.

Symptomatic seizures are common in primary and secondary brain tumors.^1^ In case of glioblastoma, 1 of 4 individuals shows seizures at onset (SAO) of disease as first clinical symptom.^2^ In addition, glioblastoma patients frequently develop secondary epilepsy due to rapid tumor progression.^3^

Several previous reports have addressed the association between SAO occurrence and glioblastoma survival, both with positive^2,4–8^ and negative^9–17^ results. The majority of recent studies has specifically focused on the role of antiepileptic drugs (AED) on the survival effect of SAO.^6,12,18–24^ However, a recent large pooled analysis of prospective clinical trials^25^ has failed to show any survival benefit from the use of AED in glioblastoma.

In this context, the knowledge of SAO predictors might be essential for a better understanding of these early glioblastoma-associated seizures and possible links with patients survival. Unfortunately, evidence on SAO predictors in glioblastoma is sparse, since the majority of SAO-related studies is based on either heterogenous (high and low grade) glioma cohorts or small glioblastoma populations.^6,14,26,27^ Even less is known on the rate, risk factors and clinical impact of early postoperative seizures (EPS), which are not related to tumor progression and therefore have unclear pathophysiologic background.

Accordingly, this study analyzed the risk factors for SAO and the relationship of SAO with overall survival (OS) in a large consecutive glioblastoma cohort. Moreover, we addressed the predictors and the clinical impact of EPS after glioblastoma surgery, as well as the similarities and differences between SAO and EPS.

## Materials and Methods

### Patient Population and Clinical Management

All consecutive cases with newly diagnosed glioblastoma treated between January 2006 and December 2018 in our institution were eligible for this study. The exclusion criteria were the following: (a) pediatric cases (<18 years old), (b) previous history of epilepsy, and (c) extracranial location. The retrospective study was conducted in accordance with the STROBE guidelines and was approved by the Institutional Ethics Committee, University of Duisburg-Essen (15-6504-BO).

All cases were histologically confirmed via stereotactic biopsy or tumor resection. Early postoperative MRI within 72 h after surgery was performed after tumor resection. Standard chemoradiation with concomitant and adjuvant temozolomide^28^ was initiated after surgery. Patients with poor perioperative neurological condition and/or without willingness for further treatment, were referred to best supportive care.

According to the current guidelines,^29^ AED treatment was usually initiated after the first seizure event. Prophylactic use of AED was not routinely performed in the cohort, except for selected cases.

### Data Management

The following patients characteristics were collected from the electronic medical records: demographic (age and sex) and anthropometric parameters (body height, weight, and body mass index), medical history (arterial hypertension, diabetes mellitus, history of cancer, and hypothyroidism), KPS score at admission, tumor location, extent of resection (EOR), immunohistochemical and molecular-genetic parameters (expression of glial fibrillary acidic protein [GFAP], p53 and Ki-67 proliferation index, the isocitrate dehydrogenase 1 gene [IDH1] mutation, and O^6^-methylguanine DNA methyltransferase [MGMT] promoter methylation status). In addition, 27 routine laboratory measurements at admission and 3 surrogate markers were assessed (see the Supplementary Table S1 with the full list of over 100 variables correlated with the study endpoints). Finally, overall survival or date of the last outpatient follow-up was assessed.

The evaluation of histological specimens was based on the original reports of 2 clinical neuropathologists, as described previously.^30^ All histological and molecular findings were reviewed in accordance with the 2016 Classification of the Central Nervous System Tumors of the World Health Organization.^31^ Tumor location was assessed upon the review of the preoperative MRI imaging. In addition, postoperative MRI scans were analyzed with regard to EOR. The cases without contrast-enhancing residual after tumor resection were considered gross-total resection (GTR), the remaining cases were regarded as tumor debulking. All available preoperative and postoperative MRI scans were reviewed by the first author (Y.A.) blinded at this time for any clinical information.

The diagnosis of epilepsy was based on the occurrence of clinical symptoms suspicious for seizures (involuntary movements, abnormal sensory signs, or an altered mental status). Additionally, patients underwent an electroencephalogram in case of questionable/nonconclusive clinical symptoms. All patients with symptomatic epilepsy were consulted by in-house epileptologists, for diagnosis confirmation, assessment of seizure semiology, and medical treatment. Epileptic seizures leading to the radiographic diagnosis of glioblastoma were regarded as SAO. Postoperative epileptic seizures occurring up to 3 weeks after surgery and prior to the begin of chemoradiation were regarded as EPS. When available, semiology (secondary generalized, simple or complex focal, or status epilepticus) and timing (in relation to the surgery day) of the seizures were documented. The hospital records were also reviewed for AED treatment.

### Study Endpoints and Statistical Analysis

The following primary endpoints were addressed in this study: (a) independent predictors of SAO and (b) EPS and (c) association between the SAO/EPS and OS. The secondary endpoint of the study was the evaluation of the patients’ characteristics related to the seizure semiology.

Statistical analyses were performed with the help of PRISM (version 5.0, GraphPad Software Inc.) and SPSS (version 25, SPSS Inc., IBM). Patients’ baseline characteristics were expressed as mean ± standard deviation (SD) or percentage of patients, as appropriate. For OS data, median values with interquartile range were reported. Differences with a *P* ≤ .05 were regarded as statistically significant.

First, all associations between the potential risk factors and the study endpoints were tested using univariate analysis. For univariate correlations, differences between continuous variables were analyzed using the Student’ *t*-test for normally distributed data and the Mann–Whitney *U* test for non-normal distributed data; associations between categorical variables were analyzed using the chi-square or Fisher’s exact tests, as appropriate.

Laboratory measurements were evaluated as continuous variables and in dichotomized manner, according to the common reference values for upper and lower ranges. For laboratory tests showing significant associations with the endpoints only as continuous variables, an additional dichotomization was performed upon the cutoffs defined on the receiver operating characteristic (ROC) curve. For immunohistochemical tumor characteristics, the dichotomization was applied using the cutoffs reported previously in the literature and/or the results from the ROC curves.

The significant correlations from univariate analyses were then evaluated in a multivariate analysis. Binary logistic regression analysis was used for the identification of independent predictors of SAO/EPS, as well as for their relation to EOR. Laboratory markers were first tested in a separate multivariate assessment prior to the inclusion in the final regression analysis. For correlation between SAO/EPS and OS, a Cox proportional-hazards model was applied by adding the common outcome confounders (age, KPS, EOR, molecular markers, and adjuvant treatment). Missing data were replaced using multiple imputation.

### Data Availability Statement

Any data not published within the article will be shared in anonymized manner by request from any qualified investigator.

## Results

### Patient Population

After the exclusion of noneligible cases (age < 18 years, *n* = 7; history of epilepsy, *n* = 6; and spinal glioblastoma, *n* = 1), 867 individuals were included in the final analysis. The baseline characteristics of the cohort are presented in Table 1.

**Table 1.** Baseline Characteristics of 867 Glioblastoma Patients Included in the Final Analysis

Parameter	*N* (%) or Mean (±SD)
Age, years	63.83 (±11.51)
Sex, male	501 (57.8%)
Medical history	
Arterial hypertension	450 (51.9%)
Diabetes mellitus	150 (17.3%)
Hypothyroidism	104 (12.0%)
History of cancer	127 (14.6%)
KPS at admission^a^	
Good (90–100%)	338 (39.7%)
Reduced (70–80%)	414 (48.6%)
Poor (≤60%)	100 (11.7%)
EOR, biopsy	247 (28.5%)
Tumor location	
Frontal lobe	305 (35.2%)
Temporal lobe	239 (27.6%)
Parietal lobe	103 (11.9%)
Occipital lobe	129 (14.9%)
Midline/Infratentorial/Bi-hemispheric	91 (10.5%)
Molecular status	
MGMT methylation	310 (41.6%^b^)
IDH1 mutation	17 (3.1%^c^)

EOR, extent of resection; IDH1-Isocitrate dehydrogenase 1 mutation; MGMT, O[6]-methylguanine-DNA methyltransferase promoter methylation; *N*, number of cases; SD, standard deviation.

^a^15 cases (1.7%) with missing KPS score.

^b^121 cases (14%) with missing MGMT promoter methylation status.

^c^311 cases (35.9%) with missing IDH1 mutation status.

### Perioperative Seizures: Occurrence and Management

The rates of SAO and EPS in the cohort were 27.2% (*n* = 236) and 7.7% (*n* = 67), respectively. Due to initially minor radiographic findings, a watch-and-wait strategy was applied in 14 SAO patients, resulting in late surgery between 2 and 11 months after seizure onset (mean interval: 5.71 months). In the remaining cases, all patients (with or without SAO) were operated within 2 weeks after the radiographic diagnosis. EPS were documented on the mean postoperative day 4.6 (±5.0). Twenty-three glioblastoma patients developed >1 separate seizure event during the perioperative course. Of them, 10 patients showed SAO and EPS.

AED treatment was initiated after the occurrence of first seizure(s) in all cases except for 13 individuals with SAO. In addition, 13 patients received prophylactic AED treatment without preceding seizures. There was a wide range of AED used in the cohort, with levetiracetam and valproic acid as the most common drugs.

### Predictors of Perioperative Seizures

**Figure 1.**
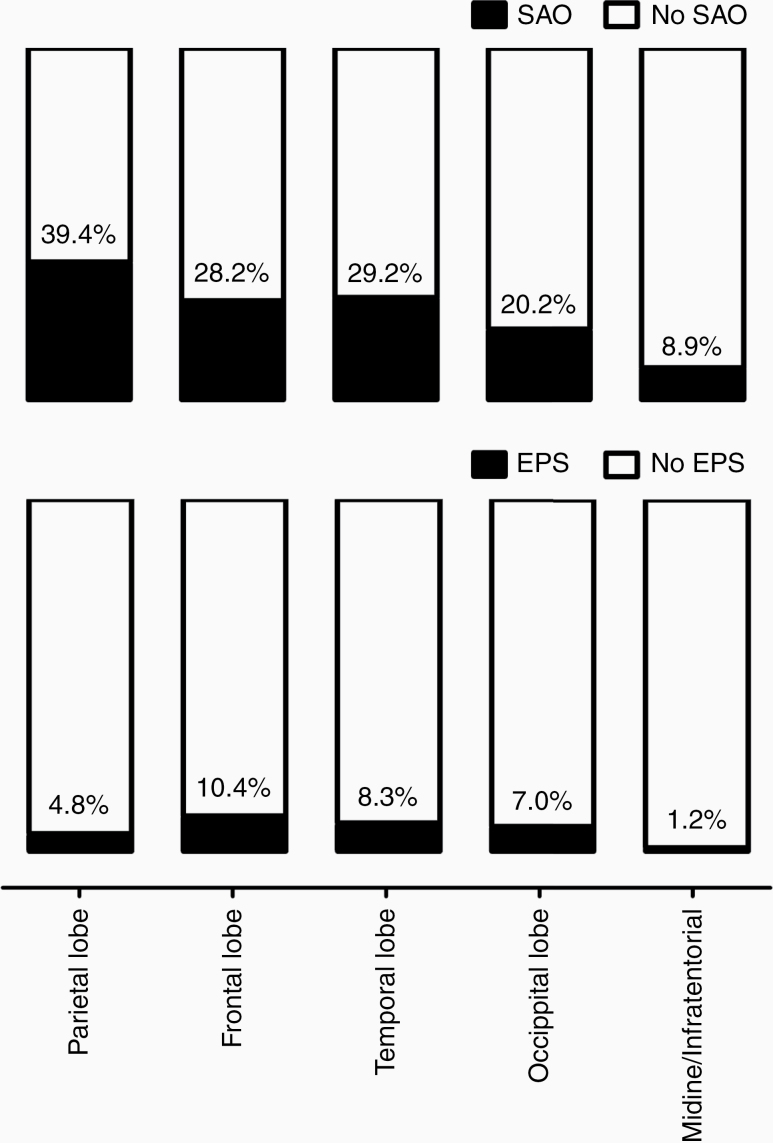
Incidence of SAO and EPS depending on tumor location

SAO and EPS showed partially contrary correlations with the baseline characteristics (see Table 2 for the univariate analyses of the results reaching the significance level for at least one study endpoint, for the full list of all associations see the Supplementary Table S1). In particular, SAO were more common in individuals with younger age (*P* = .001) and higher preoperative KPS score (*P* < .0001). The tumors located in the parietal lobe (odds ratio [OR]: 1.96, *P* = .003, see Figure 1 for the distribution of SAO/EPS rates in different brain areas), those with higher GFAP expression (≥35%, OR: 1.88, *P* = .05) and more radical EOR (tumor resection vs biopsy [OR: 1.87, *P* = .001] and GTR vs debulking [OR: 1.64, *P* = .006]) were also associated with SAO. In turn, EPS were related to comorbidities (arterial hypertension [OR: 1.74, *P* = .040] and history of cancer [OR: 1.87, *P* = .049]) and tumors located in the frontal or temporal lobe (OR: 2.07, *P* = .017). Moreover, there was an inverse association between the EOR and occurrence of EPS in the individuals undergoing tumor resection (GTR vs debulking, OR: 0.42, *P* = .005). Finally, certain admission laboratory variables were also associated with the SAO and EPS. Of note, pathologic laboratory values were more characteristic for EPS than for SAO.

**Table 2.** Shortened List of Univariate Assessments Between the Patients’ Characteristics and the Occurrence of SAO and EPS (for the full list with over 100 tested variables—see the Supplementary Table S1)

Parameter	SAO		EPS	
	OR (95% CI)/mean (±SD)	*P*-value	OR (95% CI)/mean (±SD)	*P*-value
Age	61.24 (±12.16) vs 64.21 (±12.42)	**.001**	63.62 (±13.49) vs 63.39 (±12.32)	.677
Arterial hypertension	0.94 (0.69–1.27)	.695	1.74 (1.02–2.96)	**.040**
History of cancer	0.72 (0.46–1.14)	.193	1.87 (1.03–3.40)	**.049**
Location: parietal lobe	1.96 (1.28–3.01)	**.003**	0.57 (0.22–1.44)	.327
Location: frontal/temporal lobe	1.28 (0.93–1.76)	.149	2.07 (1.15–3.75)	**.017**
EOR: Surgery vs Biopsy	1.87 (1.30–2.68)	**.001**	1.72 (0.92–3.21)	.092
EOR: GTR vs Debulking	1.64 (1.15–2.35)	**.006**	0.42 (0.23–0.76)	**.005**
GFAP staining. ≥ 35%	1.88 (1.02–3.47)	**.053**	0.91 (0.37–2.28)	.812
KPS % continuous	83.16 (±12.19) vs 78.67 (±13.18)	**<.0001**	78.09 (±13.74) vs 80.09 (±12.99)	.304
Erythrocyte < 4.0/pL	2.12 (0.99–4.55)	.079	4.28 (1.74–10.48)	**.004**
WBC > 11/nL	0.40 (0.28–0.55)	**<.0001**	1.09 (0.66–1.82)	.796
Hemoglobin < 12 g/dL	1.43 (0.75–2.72)	.285	3.34 (1.53–7.29)	**.005**
Hematocrit < 35%	1.44 (0.70–2.94)	.332	3.16 (1.33–7.55)	**.015**
PLT > 400/nL	0.32 (0.11–0.90)	**.022**	0.32 (0.04–2.34)	.352
Sodium < 140 mmol/L	0.67 (0.49–0.94)	**.019**	1.17 (0.70–1.97)	.591
Chloride > 102 mmol/L	1.72 (1.22–2.42)	**.002**	0.84 (0.50–1.43)	.581
Calcium > 2.35 mmol/L	1.75 (1.24–2.46)	**.002**	0.49 (0.26–0.95)	**.039**
Urea > 20 mg/dL	0.53 (0.39–0.73)	**<.0001**	1.62 (0.97–2.71)	.068
GGT > 55 U/L	0.99 (0.67–1.47)	1.000	2.19 (1.26–3.81)	**.008**
Total protein <7.0 g/dL	0.67 (0.48–0.92)	**.014**	1.83 (1.05–3.18)	**.033**
CRP >=1.0 mg/dL	0.96 (0.58–1.59)	.898	3.00 (1.39–6.51)	**.006**
PLT/WBC > 25	1.64 (1.20–2.24)	**.002**	0.82 (0.50–1.38)	.519
WBC/MPV < 0.9	2.69 (1.87–3.88)	**<.0001**	1.12 (0.63–2.00)	.768

CRP, C-reactive protein; EOR, extent of resection; GFAP, glial fibrillary acidic protein immunohistochemistry staining percentage; GGT, gamma-glutamyl-transferase; GTR, gross total resection; MPV, mean platelet volume; OR, odds ratio; PLT, blood platelets; SD, standard deviation; WBC, white blood cells. Differences with a P ≤ .05 were regarded as statistically significant.

In the multivariate analysis (see Table 3 and Supplementary Table S2), SAO were independently associated with younger age (*P* = .009), higher KPS (*P* = .002), tumor location (parietal lobe, *P* = .001), higher GFAP expression (≥35%, *P* = .045), and serum chloride levels (>102 mmol/L, *P* = .004). In turn, EPS were independently associated with tumor location (frontal or temporal lobe, *P* = .013) and the following admission laboratory parameters: increased c-reactive protein (>1.0 mg/dL, *P* = .036) and Gamma-Glutamyl-Transferase levels (>55 U/L, *P* = .025), and presence of anemia (hemoglobin < 12 g/dL, *P* = .044/red blood cells count < 4/nL, *P* = .041, see also Supplementary Table S3 for the appropriate multivariate analysis).

**Table 3.** Multivariate Analysis of Independent Predictors of SAO and EPS

Parameters	aOR (95% CI)	*P*-value
Predictors of SAO		
Location: parietal lobe	2.13 (1.37–3.33)	**.001**
Age, years	0.98 (0.97–0.99)	**.009**
KPS score, %	1.02 (1.01–1.04)	**.002**
IHC: GFAP ≥35%	2.07 (1.02–4.20)	**.045**
Blood test: Chloride >102 U/L	1.68 (1.18–2.39)	**.004**
Blood test: Calcium >2.35 mmol/L	1.29 (0.87–1.93)	.206
Predictors of EPS		
Location: frontal/temporal lobe	2.34 (1.20–4.56)	**.013**
Comorbidity: Arterial hypertension	1.52 (0.86–2.68)	.154
Comorbidity: Second malignancy	1.62 (0.85–3.07)	.144
Blood test: Hemoglobin <12 g/dL	2.32 (1.02–5.24)	**.044**
Blood test: GGT >55 U/L	1.94 (1.09–3.47)	**.025**
Blood test: CRP >1.0 mg/dL	2.45 (1.07–5.58)	**.036**

aOR, adjusted odds ratio; CRP, C-reactive protein; IHC, immunohistochemistry; GFAP, glial fibrillary acidic protein staining percentage; GGT, gamma-glutamyl-transferase. Differences with a P ≤ .05 were regarded as statistically significant.

### Impact of Perioperative Seizures on OS

**Figure 2.**
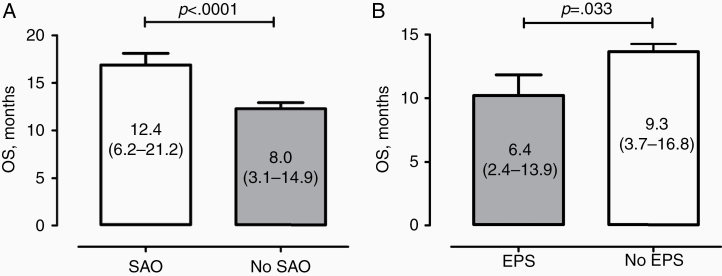
OS (median values including the interquartile range in parentheses) of the patients in the cohort depending on the occurrence of SAO (A) or EPS (B) in the perioperative course.

Correlation between perioperative seizures and patients’ survival showed contrary effects of SAO and EPS. In particular, OS was significantly longer in individuals with SAO than without (12.4 vs 8.0 months, *P* < .0001, Figure 2A). In contrast, patients with EPS had poorer outcome, as compared to the counterparts without EPS (6.4 vs 9.3 months, *P* = .033, Figure 2B). Of note, glioblastoma individuals with SAO without surgery delay showed a trend to longer OS than the above-mentioned 14 cases with delayed surgery after SAO (13.0 vs 7.7 months, *P* = .1073).

**Figure 3.**
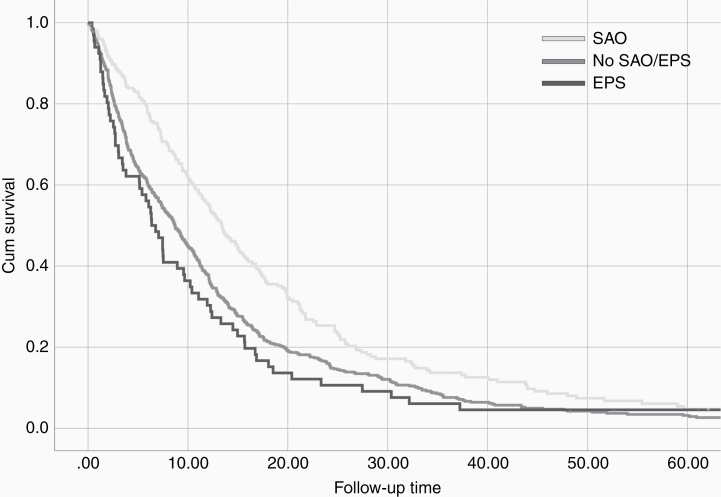
Kaplan–Meier survival plot showing the survival differences between the patients with SAO, EPS, and no perioperative seizures (no SAO and no EPS).

The multivariate analysis for independent OS predictors (adjusted for patients’ age, KPS, EOR, MGMT/IDH1 status, and postoperative chemoradiation, see Supplementary Table S4) confirmed the positive impact of SAO (adjusted hazard ratio [aHR]: 0.82, 95% confidence interval [CI]: 0.69–0.98, *P* = .03) and negative impact of EPS (aHR: 1.41, 95% CI: 1.09–1.83, *P* = .009) on the OS. Kaplan–Meier Survival plot (Figure 3) shows different survival curves in the cohort depending on the presence or absence of SAO and EPS.

### Seizure Semiology

Of the cases with documented seizure type (*n* = 262/293), patients developed more frequently secondary generalized seizures (*n* = 134, 51.1%) than simple (*n* = 114, 43.5%) or complex focal (*n* = 13, 5%) convulsions. One patient (0.4%) presented with epileptic status at disease onset. Individuals with secondary generalized seizures were significantly younger, than the patients with other seizure types (59.57 [±11.55] vs 63.74 [±11.38] years, *P* = .003). Remaining patients’ characteristics showed no significant associations with the seizure semiology. Finally, there was a trend toward a longer OS in case of secondary generalized seizures (12.3 vs 10.5 months, *P* = .074).

## Discussion

The risk factors for perioperative seizures in glioblastoma remain poorly understood. Moreover, the clinical impact of SAO and EPS is still a matter of debate. In this large consecutive series of glioblastoma patients, we have identified different risk patterns for the occurrence of SAO and EPS. In addition, there was a contrary effect of SAO and EPS on OS.

### Epileptogenesis of SAO

The studies on the predictors of SAO are mostly based on heterogenous glioma cohorts and presume higher risk of SAO (and of symptomatic seizures in general) in low-grade glioma, partially due to the survival differences compared with high-grade glioma.^1,32,33^ Recent reports pointed to the crucial role of IDH1 mutation in tumor epileptogenesis.^14,26,34,35^ Other molecular-genetic and immunohistochemical tumor markers like 1p19q-codeletion, MGMT promoter methylation, BDNF, ADK, BRAF V600E mutations, MMP-9, expressions of miR-128, nuclear protein Ki-67, p53, RINT1, and VLGR, EGFR amplification, and PI3K-mTOR pathway were also addressed, and partially linked with the risk of symptomatic epilepsy in glioma patients.^1,3,32,36,37^

As to the studies based on glioblastoma cohorts, younger age,^14,34^ certain tumor locations,^13,14^ expression of p53^34^ and glutamine synthetase,^10,15^ higher preoperative KPS score,^14^ smaller volume of tumor, intratumoral necrosis and peri-tumoral edema,^10,11^ and statin medication (inversely)^11^ were reported to be associated with SAO. At the same time, other studies did not identify any relationship between the patients’ age,^13^ tumor location,^10^ and size^13^ with the occurrence of preoperative seizures. The major limitation of all these studies refers to mostly smaller cohort size and missing evaluation of the independent predictive value of each of the reported risk factors.

Our large glioblastoma-based cohort confirmed the essential role of such predictors like younger age, higher KPS score and tumor location in the genesis of SAO. In addition, we identified 2 other independent risk factors for SAO—higher expression of GFAP in tumor tissue and high-normal to increased levels of serum chloride (>102 mmol/L). In glial cells, GFAP is involved in cytoskeleton architecture, maintaining the mechanical strength, the associations with surrounding neurons and the blood–brain barrier.^30^ The relationship between GFAP expression and seizure activity in astrocytes has been addressed in experimental epilepsy studies beyond oncological research,^38,39^ and has been described in selected cases of glioma patients.^40^ Whether higher GFAP expression leads to increased epileptogenicity or vice versa remains uncertain, since increase of GFAP expression secondary to epileptic seizures was demonstrated experimentally.^41^ Notably, higher GFAP expression in low-grade glioma was previously reported.^30,42^ This circumstance is in line with potential epileptogenic role of GFAP in glial tumors, since higher prevalence of epilepsy in low- versus high-grade glioma has already been mentioned before. In virtue of all these findings, further research of the role of GFAP in seizure activity of glial tumors is mandatory.

Moreover, we analyzed a wide range of admission laboratory values and found independent associations between higher serum chloride levels and the occurrence of SAO in glioblastoma. The interpretation of admission blood tests with regard to SAO epileptogenesis is generally problematic. The altered laboratory values might rather be secondary to seizure event(s), than provide the insights into the systemic processes, which promote the seizures. This would particularly explain higher levels of calcium and sodium in serum of individuals with SAO, because seizures can result in hypercalcemia and hypernatremia.^43^ As to higher chloride levels in SAO patients, this finding might have certain causal implications. The impact of chloride ions on seizure activity in neuronal cells is widely acknowledged and related to gamma-Aminobutyric acid (GABA) receptor activity.^44,45^ Experimental research with glial tumor cells has demonstrated that elevated intracellular chloride concentrations cause hyperpolarization of GABAergic neurons and lead to reduced network.^1,46^ In consequence, cumulative reduction of the inhibitory postsynaptic potential due to reduced GABAergic synaptic density is supposed to foster.^1,46,47^ Therefore, our findings strengthen the current hypotheses on the epileptogenesis of gliomas.

### EPS: A Strong Negative Predictor

In contrast to delayed seizures due to postoperative tumor progression, less is known about the incidence and the genesis of EPS. This is the first study addressing the risk factors for and clinical consequences of EPS after glioblastoma surgery. Unlike SAO, EPS were more related to systemic dysfunctions present at the time of admission like anemia, systemic infection, and altered liver homeostasis. In addition, previous medical history (arterial hypertension and history of cancer) was also associated with EPS risk, however only in univariate analysis. It has been reported that various systemic diseases like endocrine, electrolyte and autoimmune disorders, organ dysfunction and failure, cancer, and paraneoplastic disorders facilitate seizure activity.^48^ Therefore, the risk of EPS after glioblastoma surgery might already be estimated preoperatively allowing timely selection of the patients requiring special postoperative care.

Not less interesting is the association of EPS with EOR and OS. The link between incomplete tumor resection and postoperative seizure risk has already been reported for glial^13^ and metastatic brain^49^ tumors. In summary, poorer outcome of glioblastoma individuals with EPS might be related to incomplete tumor resection and presence of above-mentioned systemic disorders.

### Symptomatic Seizures in Glioblastoma: Is There Any Link With Outcome?

A large number of studies has addressed the association between early symptomatic seizures and glioblastoma survival reporting on longer OS in patients with SAO.^2,4–8^ Noteworthy, there are also several publications not confirming the predictive impact of SAO on patients survival.^9–17^ Moreover, many recent studies focused not on the SAO event as outcome confounder, but on the potential effect of AED on glioblastoma survival. Antitumoral activity of different AED was analyzed in numerous clinical and experimental studies.^1,12,23,33,50^ Unfortunately, the results are strongly conflicting, both with^18–21^ and without^6,12,22–25^ any association between AED use and survival. A recent large pooled analysis of prospective clinical trials^25^ has failed to show any survival benefit from the use of AED in newly diagnosed glioblastoma.

Our results might shed some light on the backgrounds of discrepant results of the previous studies on the role of AED. Occurrence of EPS also necessitates early initiation of AED treatment. In turn, EPS are associated with poorer survival after glioblastoma surgery, as we demonstrate in the present study. Therefore, future studies on AED effect on glioblastoma survival should also take the timing and causal background of perioperative seizures into account.

We could show independent association between SAO and OS. The vast majority of the studies, which failed to show a benefit of SAO on glioblastoma survival were based on relatively small cohorts and their results were therefore statistically underpowered. A recent meta-analysis^2^ also showed extended OS in individuals with SAO. In virtue of the findings of the present and previous studies, the following major conclusions regarding the SAO and OS can be made: (a) occurrence of SAO is associated with more favorable outcome of glioblastoma; (b) this effect might be related to earlier diagnosis of glioblastoma, since these patients present with higher KPS score and smaller tumor burden, and the SAO individuals with surgery delay seem to “lose” the survival benefit; (c) clinical characteristics of SAO patients like younger age and more radical EOR might also contribute to better treatment results.

### Study Limitations

Retrospective design presents the major limitation of this study. Therefore, there is certain portion of missing data due to incomplete documentation in the hospital records. Regarding the molecular features of glioblastoma, missing data is mainly related to later implementation of molecular markers into the diagnostic set-up of glioblastoma, for example, IDH1 mutation status. These variables are insofar important because molecular tumor characteristics might present the causal link explaining the association between the seizure risk and survival in glioblastoma patients. Therefore, further research on the role of already established and novel molecular tumor markers on epileptogenesis in glioblastoma are mandatory. Nevertheless, we present a study based on a large consecutive series and adjust the study results for relevant endpoint confounders and information bias using multivariate analysis and multiple imputation.

## Conclusions

In glioblastoma patients, SAO are independently associated with younger age, better preoperative clinical performance, certain tumor characteristics (location in the parietal lobe and higher GFAP expression), and serum ion alterations (higher chloride levels). In addition, SAO are related to more radical EOR and favorable OS. In contrast, EPS are strongly associated with presence of systemic disorders (anemia, infection, and liver dysfunction), incomplete tumor resection and poorer OS. Our results encourage further analysis of the effect of perioperative seizures on glioblastoma survival.

## Supplementary Material

vdaa158_suppl_Supplementary_Materials

## Notes

**Authorship Statement.** All authors designed the study, interpreted the data, critically revised and provided final approval for the manuscript, and are accountable for the accuracy of its contents. Study conception and design: R.J. and Y.A. Acquisition of data: R.J., Y.A., L.R., A.S., and L.L. Analysis and interpretation of data: R.J., Y.A., M.D., D.P., P.D., K.W., A.J., and B.S. Drafting of manuscript: R.J. and Y.A. Critical revision: C.Q., M.G., and M.S. Project administration: R.J. and U.S.
