# Supplementary material for: Preoperative and early postoperative seizures in patients with glioblastoma—two sides of the same coin?
Source: Neurooncol Adv. 2020 Nov 18;3(1):vdaa158. doi: 10.1093/noajnl/vdaa158 (PMC7813191; doi:10.1093/noajnl/vdaa158)
Supplement: vdaa158_suppl_Supplementary_Materials [file vdaa158_suppl_supplementary_materials.docx]

**SUPPLEMENTARY MATERIALS:**

**Preoperative and early postoperative seizures in patients with glioblastoma – Two sides of the same coin?**

*Yahya Ahmadipour^1,6^, MD (*[*yahya.ahmadipour@uk-essen.de*](mailto:yahya.ahmadipour@uk-essen.de)*); Laurèl Rauschenbach^1,6^, (*[*laurel.rauschenbach@uk-essen.de*](mailto:laurel.rauschenbach@uk-essen.de)*); Alejandro Santos^1^, (*[*alejandro.santos@uk-essen.de*](mailto:alejandro.santos@uk-essen.de)*); Marvin Darkwah Oppong^1,6^, MD (*[*marvin.darkwahoppong@uk-essen.de*](mailto:marvin.darkwahoppong@uk-essen.de)*); Lazaros Lazaridis^2,6^, MD (*[*lazaros.lazaridis@uk-essen.de*](mailto:lazaros.lazaridis@uk-essen.de)*); Carlos Quesada^3^, MD (*[*carlos.quesada@uk-essen.de*](mailto:carlos.quesada@uk-essen.de)*); Andreas Junker^4^, MD (*[*andreas.junker@uk-essen.de*](mailto:andreas.junker@uk-essen.de)*); Daniela Pierscianek^1,6^_,_ MD (*[*daniela.pierscianek@uk-essen.de*](mailto:daniela.pierscianek@uk-essen.de)*); Philipp Dammann^1,6^, MD (*[*philipp.dammann@uk-essen.de*](mailto:philipp.dammann@uk-essen.de)*); Karsten H. Wrede^1,6^, MD (*[*karsten.wrede@uk-essen.de*](mailto:karsten.wrede@uk-essen.de)*); Björn Scheffler^1,6^, MD (*[*Bjoern.Scheffler@uk-essen.de*](mailto:Bjoern.Scheffler@uk-essen.de)*); Martin Glas^2,6^, MD (*[*martin.glas@uk-essen.de*](mailto:martin.glas@uk-essen.de)*); Martin Stuschke^5,6^, MD (*[*martin.stuschke@uk-essen.de*](mailto:martin.stuschke@uk-essen.de)*); Ulrich Sure^1,6^, MD (*[*ulrich.sure@uk-essen.de*](mailto:ulrich.sure@uk-essen.de)*); Ramazan Jabbarli^1,6^, MD (*[*ramazan.jabbarli@uk-essen.de*](mailto:ramazan.jabbarli@uk-essen.de)*)*

1 - Department of Neurosurgery and Spine Surgery, University Hospital Essen, Germany.

2 - Division of Clinical Neurooncology, Clinic for Neurology, University Hospital Essen, Germany.

3 - Clinic for Neurology, University Hospital Essen, Germany.

4 - Department of Neuropathology, University Hospital Essen, Germany.

5 - Department of Radiotherapy, University Hospital Essen, Germany.

6 - German Cancer Consortium, Partner Site University Hospital Essen, Essen, Germany

**CORRESPONDING AUTHOR**: Ramazan Jabbarli. MD

Department of Neurosurgery and Spine Surgery

University Hospital of Essen.

D-45147 Essen. Germany

E-Mail: [ramazan.jabbarli](mailto:ramazan.jabbarli)@uk-essen.de

Phone: +49 201 723 1201

Fax: +49 201 723 5991

**Table S1:** Full list of 110 parameters of univariate assessments and the occurrence of SAO and EPS

| **Parameter** | **SAO** | | **EPS** | |
| --- | --- | --- | --- | --- |
|  | **OR (95% CI) / mean (±SD)** | **p-value** | **OR (95% CI) / mean (±SD)** | **p-value** |
| Sex. female | 0.81 (0.60 - 1.10) | 0.190 | 1.20 (0.73 - 1.97) | 0.521 |
| Age | 61.24 (±12.16) vs 64.21 (±12.42) | **0.001** | 63.62 (±13.49) vs 63.39 (±12.32) | 0.677 |
| Age <63 years | 1.61 (1.19 - 2.17) | **0.002** | 0.89 (0.54 - 1.48) | 0.702 |
| Age <64 years | 1.59 (1.18 - (2.16) | **0.003** | 0.91 (0.55 - 1.49) | 0.705 |
| Age <65 years | 1.55 (1.15 - 2.10) | **0.005** | 0.88 (0.53 - 1.45) | 0.703 |
| Body height. cm | 173.96 (±9.93) vs 172.51 (11.24) | 0.062 | 173.88 (±11.42) vs 172.84 (±10.86) | 0.690 |
| Body weight. kg | 82.23 (±15.53) vs 80.69 (±17.05) | 0.172 | 82.15 (±18.02) vs 81.03 (±16.53) | 0.813 |
| BMI. kg/m2 | 27.03 (±4.88) vs 26.93 (±4.65) | 0.944 | 26.53 (±4.89) vs 26.99 (±4.70) | 0.713 |
| Arterial hypertension | 0.94 (0.69 - 1.27) | 0.695 | 1.74 (1.02 - 2.96) | **0.040** |
| Diabetes mellitus | 0.82 (0.62 - 1.38) | 0.761 | 1.78 (1.00 - 3.16) | 0.065 |
| History of cancer | 0.72 (0.46 - 1.14) | 0.193 | 1.87 (1.03 - 3.40) | **0.049** |
| Hypothyroidism | 0.88 (0.55 - 1.41) | 0.640 | 1.15 (0.55 - 2.40) | 0.695 |
| Tumor location: left vs right hemisphere | 1.25 (0.92 - 1.69) | 0.162 | 1.08 (0.65 - 1.81) | 0.794 |
| Tumor location: parietal lobe vs other location | 1.96 (1.28 - 3.01) | **0.003** | 0.57 (0.22 - 1.44) | 0.327 |
| Tumor location: frontal/temporal lobe vs. other location | 1.28 (0.93 -1.76) | 0.149 | 2.07 (1.15 - 3.75) | **0.017** |
| Tumor location: >1 lobe involved | 0.99 (0.69 - 1.41) | 1.000 | 1.39 (0.80 - 2.43) | 0.286 |
| Extent of resection: Surgery vs Biopsy | 1.87 (1.30 - 2.68) | **0.001** | 1.72 (0.92 - 3.21) | 0.092 |
| Extent of resection: GTR vs Subtotal | 1.64 (1.15 - 2.35) | **0.006** | 0.42 (0.23 - 0.76) | **0.005** |
| GFAP staining. % (continuous) | 63.91 (±21.99) vs 62.26 (±26.16) | 0.680 | 59.0 (±26.34) vs 62.96 (±25.05) | 0.393 |
| GFAP staining. ≥25% | 1.84 (0.84 - 4.02) | 0.168 | 1.21 (0.36 - 4.07) | 1.000 |
| GFAP staining. ≥35% | 1.88 (1.02 - 3.47) | 0.053 | 0.91 (0.37 - 2.28) | 0.812 |
| p53 staining. % (continuous) | 12.54 (±20.19) vs 15.20 (±23.40) | 0.274 | 14.69 (±21.08) vs 14.48 (±22.76) | 0.450 |
| p53 staining. >5% | 0.80 (0.55 - 1.17) | 0.259 | 0.95 (0.52 - 1.74) | 1.000 |
| p53 staining. >10% | 0.85 (0.56 - 1.27) | 0.473 | 1.14 (0.60 - 2.15) | 0.739 |
| Ki-67-index. % (continuous) | 19.72 (±11.71) vs 19.63 (±11.90) | 0.846 | 21.46 (±15.85) vs 19.50 (±11.44) | 0.943 |
| Ki-67-index. >10% | 1.20 (0.79 - 1.83) | 0.463 | 1.03 (0.52 - 2.03) | 1.000 |
| Ki-67-index. >20% | 1.04 (0.70 - 1.55) | 0.919 | 0.98 (0.51 - 1.90) | 1.000 |
| MGMT | 1.02 (0.73 - 1.41) | 0.934 | 0.99 (0.59 - 1.67) | 1.000 |
| IDH1 | 1.62 (0.59 - 4.45) | 0.397 | 1.46 (0.32 - 6.60) | 0.647 |
| KPS. % (continuous) | 83.16 (±12.19) vs 78.67 (±13.18) | **<0.0001** | 78.09 (±13.74) vs 80.09 (±12.99) | 0.304 |
| KPS. ≤60% | 0.74 (0.44 - 1.24) | 0.322 | 1.83 (0.94 - 3.56) | 0.121 |
| KPS. ≤70% | 0.55 (0.38 - 0.79) | **0.001** | 1.60 (0.95 - 2.69) | 0.121 |
| KPS. ≤80% | 0.50 (0.36 - 0.68) | **<0.0001** | 0.96 (0.57 - 1.59) | 0.896 |
| Erythrocyte (/pl) | 4.77 (±0.50) vs 4.78 (±0.48) | 0.966 | 4.63 (±0.66) vs 4.79 (±0.47) | 0.087 |
| Erythrocyte <4.0 (/pl) | 2.12 (0.99 - 4.55) | 0.079 | 4.28 (1.74 - 10.48) | **0.004** |
| WBC (/nl) | 10.38 (±8.49) vs 11.67 (±4.57) | **<0.0001** | 11.19 (±5.27) vs 11.34 (±5.96) | 0.634 |
| WBC <4.5 (/nl) | 1.93 (0.72 - 5.12) | 0.179 | 0.73 (0.10 - 5.58) | 1.000 |
| WBC >11 (/nl) | 0.40 (0.28 - 0.55) | **<0.0001** | 1.09 (0.66 - 1.82) | 0.796 |
| Hemoglobin (g/dl) | 14.35 (±1.45) vs 14.33 (±1.53) | 0.765 | 13.93 (±1.96) vs 14.37 (±1.46) | 0.058 |
| Hemoglobin <12 (g/dl) | 1.43 (0.75 - 2.72) | 0.285 | 3.34 (1.53 - 7.29) | **0.005** |
| Hematocrit (l/l) | 0.42 (±0.04) vs 0.48 (±1.61) | 0.359 | 0.41 (±0.05) vs 0.47 (±1.44) | 0.057 |
| Hematocrit <0.350 (l/l) | 1.44 (0.70 - 2.94) | 0.332 | 3.16 (1.33 - 7.55) | **0.015** |
| Hematocrit >0.500 (l/l) | 1.24 (0.43 - 3.60) | 0.776 | 0.78 (0.10 - 5.99) | 1.000 |
| MCV (fl) | 88.73 (±4.44) vs 87.99 (±4.89) | 0.084 | 88.21 (±4.53) vs 88.19 (±4.80) | 0.769 |
| MCV <81 (fl) | 0.26 (0.61 - 1.14) | 0.055 | 1.18 (0.27 - 5.18) | 0.688 |
| MCV >100 (fl) | 1.63 (0.39 - 6.89) | 0.451 | 1.69 (0.20 - 13.91) | 0.479 |
| MCH (pg) | 30.16 (±1.60) vs 30.04 (±1.80) | 0.812 | 30.11 (±1.60) vs 30.07 (±1.76) | 0.891 |
| MCH <26 (pg) | 0.44 (0.05 - 3.71) | 0.681 | 0.76 (0.04 - 13.52) | 1.000 |
| MCH >34 (pg) | 1.81 (0.30 - 10.92) | 0.616 | 2.96 (0.33 - 26.89) | 0.335 |
| MCHC (g/dl) | 34.0 (±1.16) vs 34.15 (±1.26) | 0.157 | 34.15 (±1.23) vs 34.11 (±1.23) | 0.930 |
| MCHC <32 (g/dl) | 1.11 (0.54 - 2.28) | 0.852 | 1.00 (0.30 - 3.36) | 1.000 |
| MCHC >36 (g/dl) | 0.40 (0.15 - 1.03) | 0.060 | 1.40 (0.48 - 4.09) | 0.531 |
| PLT (/nl) | 250.68 (±64.38) vs 268.30 (±76.96) | **0.002** | 254.43 (±74.31) vs 264.33 (±74.15) | 0.361 |
| PLT <150/nl | 0.57 (0.23 – 1.39) | 0.242 | 2.11 (0.79 – 5.64) | 0.179 |
| PLT >400/nl | 0.32 (0.11 - 0.90) | **0.022** | 0.32 (0.04 - 2.34) | 0.352 |
| MPV (fl) | 10.79 (±0.94) vs 10.70 (±0.94) | 0.289 | 10.65 (±0.96) vs 10.73 (±0.94) | 0.541 |
| MPV >12 | 1.55 (0.89 - 2.72) | 0.127 | 0.78 (0.27 - 2.23) | 0.807 |
| PT. % (continuous) | 98.65 (±10.05) vs 95.99 (±15.72) | 0.524 | 94.29 (±16.86) vs 96.85 (±14.38) | 0.542 |
| aPTT. seconds (continuous) | 25.57 (±2.30) vs 25.36 (±2.72) | 0.184 | 25.83 (±2.82) vs 25.37 (±2.60) | 0.426 |
| Sodium (mmol/l) | 140.76 (±3.29) vs 139.97 (±3.58) | **0.002** | 139.94 (±3.66) vs 140.20 (±3.51) | 0.497 |
| Sodium <140mmol/l | 0.67 (0.49 - 0.94) | **0.019** | 1.17 (0.70 - 1.97) | 0.591 |
| Hyponatremia. <135 mmol/l | 0.81 (0.43 - 1.55) | 0.640 | 1.48 (0.61 - 3.59) | 0.431 |
| Hypernatriema. >145 mmol/l | 1.21 (0.59 - 2.51) | 0.570 | 0.69 (0.16 - 2.95) | 1.000 |
| Potassium(mmol/l) | 4.31 (±0.43) vs 4.26 (±0.42) | 0.147 | 4.25 (±0.36) vs 4.28 (±0.43) | 0.607 |
| Hypokalemia. <3.4 mmol/l | 1.37 (0.41 - 4.60) | 0.743 | 0.45 (0.03 - 7.98) | 0.614 |
| Hyperkalemia. >5.2 mmol/l | 1.18 (0.45 - 3.10) | 0.799 | 0.62 (0.08 - 4.73) | 1.000 |
| Chloride (mmol/l) | 104.10 (±3.71) vs 103.33 (±3.91) | **0.011** | 103.14 (±3.93) vs 103.57 (±3.87) | 0.308 |
| Chloride >102 mmol/l | 1.72 (1.22 - 2.42) | **0.002** | 0.84 (0.50 - 1.43) | 0.581 |
| Hypochloridemia. <95 mmol/l | 0.51 (0.15 - 1.76) | 0.430 | 1.44 (0.32 - 6.36) | 0.650 |
| Hyperchloridemia. >112 mmol/l | 0.92 (0.10 - 8.86) | 1.000 | 1.33 (0.07 - 24.95) | 1.000 |
| Calcium (mmol/l) | 2.33 (±0.14) vs 2.30 (±0.14) | **0.005** | 2.27 (±0.17) vs 2.31 (±0.14) | **0.042** |
| Calcium. >2.35 mmol/l | 1.75 (1.24 - 2.46) | **0.002** | 0.49 (0.26 - 0.95) | **0.039** |
| Hypocalciemia. <2.15 mmol/l | 0.72 (0.43 - 1.22) | 0.255 | 1.76 (0.87 - 3.55) | 0.140 |
| Hypercalciemia. >2.65 mmol/ | 3.29 (0.88 - 12.40) | 0.151 | 1.44 (0.18 - 11.70) | 0.532 |
| Creatinine (mg/dl) | 1.06 (±0.24) vs 1.06 (±0.23) | 0.869 | 1.10 (±0.29) vs 1.05 (±0.23) | 0.182 |
| Creatinine. >1.2 mg/dl | 1.11 (0.76 - 1.64) | 0.618 | 1.47 (0.81 - 2.67) | 0.244 |
| Urea (mg/dl) | 18.86 (±7.89) vs 22.33 (±11.19) | **<0.0001** | 22.39 (±10.53) vs 21.31 (±10.51) | 0.266 |
| Urea >20 mg/dl | 0.53 (0.39 - 0.73) | **<0.0001** | 1.62 (0.97 - 2.71) | 0.068 |
| Urea >46 mg/dl | 0.17 (0.02 - 1.25) | 0.053 | 0.74 (0.10 - 5.67) | 1.000 |
| Bilirubin total (mg/dl) | 0.59 (±0.30) vs 0.60 (±0.33) | 0.638 | 0.56 (±0.30) vs 0.60 (±0.33) | 0.181 |
| Bilirubin total >1.0 mg/dl | 1.32 (0.73 - 2.36) | 0.352 | 0.66 (0.20 - 2.17) | 0.613 |
| Bilirubin-direct (mg/dl) | 0.20 (±0.11) vs 0.19 (±0.10) | 0.609 | 0.19 (±0.10) vs 0.20 (±0.11) | 0.788 |
| Bilirubin-direct >0.3 mg/dl | 1.31 (0.61 - 2.79) | 0.546 | 0.35 (0.05 - 2.66) | 0.498 |
| ASAT(U/l) | 29.46 (±53.90) vs 23.14 (±18.53) | 0.707 | 24.42 (±16.35) vs 24.88 (±33.19) | 0.516 |
| ASAT >50 | 2.02 (0.97 - 4.18) | 0.064 | 1.82 (0.62 - 5.37) | 0.291 |
| ALT(U/l) | 39.43 8+41.64) vs 38.14 (±41.06) | 0.628 | 41.28 (±32.33) vs 38.25 (±41.86) | 0.246 |
| ALT >45 | 1.10 (0.76 - 1.59) | 0.633 | 1.50 (0.85 - 2.66) | 0.202 |
| ALP (U/l) | 73.50 (±24.64) vs 71.82 (±25.94) | 0.225 | 77.0 (±26.08) vs 71.87 (±25.53) | 0.160 |
| ALP <35 U/l | 0.91 (0.09 - 8.85) | 1.000 | 3.95 (0.40 - 39.23) | 0.283 |
| ALP >104 U/l | 0.77 (0.30 - 1.96) | 0.659 | 2.17 (0.70 - 6.74) | 0.255 |
| GGT (U/l) | 49.55 (±88.06) vs 44.72 (±56.51) | 0.331 | 68.39 (±104.81) vs 44.16 (±61.95) | **0.006** |
| GGT>55 | 0.99 (0.67 - 1.47) | 1.000 | 2.19 (1.26 - 3.81) | **0.008** |
| LDH (U/l) | 219.43 (±60.78) vs 227.78 (±115.13) | 0.356 | 217.53 (±48.46) vs 226.21 (±106.74) | 0.867 |
| LDH >247 U/l | 0.95 (0.65 - 1.38) | 0.849 | 1.06 (0.58 - 1.95) | 0.875 |
| Total Protein (g/dl) | 6.94 (±0.64) vs 6.84 (±0.67) | **0.030** | 6.67 (±0.78) vs 6.88 (±0.64) | **0.030** |
| Total Protein <7.0 | 0.67 (0.48 - 0.92) | **0.014** | 1.83 (1.05 - 3.18) | **0.033** |
| Total Protein <6.5 | 0.79 (0.54 - 1.15) | 0.223 | 1.64 (0.94 - 2.86) | 0.090 |
| CRP (mg/dl) | 0.88 (±1.25) vs 1.36 (±2.61) | 0.502 | 1.59 (±1.74) vs 1.19 (±2.35) | **0.009** |
| CRP >0.5 | 1.04 (0.65 - 1.67) | 0.905 | 2.99 (1.24 - 7.19) | **0.012** |
| CRP >=1.0 | 0.96 (0.58 - 1.59) | 0.898 | 3.00 (1.39 - 6.51) | **0.006** |
| GFR (ml/min/1.73qm) | 70.98 (±20.73) vs 67.84 (±15.75) | 0.059 | 65.86 (±18.46) vs 68.9 (±17.13) | 0.131 |
| GFR <90 | 0.67 (0.36 - 1.26) | 0.229 | 0.90 (0.31 - 2.62) | 0.778 |
| GFR <60 | 0.74 (0.50 - 1.11) | 0.168 | 1.69 (0.93 - 3.05) | 0.104 |
| Glucose (mmol/l) | 6.63 (±2.55) vs 7.03 (±3.10) | 0.550 | 7.52 (±3.66) vs 6.89 (±2.93) | 0.611 |
| APRI | 0.26 (±0.49) vs 0.19 (±0.13) | 0.103 | 0.21 (±0.15) vs 0.21 (±0.29) | 0.276 |
| APRI >1.0 | 3.66 (0.81 - 16.47) | 0.089 | 0.78 (0.04 - 13.78) | 1.000 |
| PLT/WBC | 29.41 (±13.61) vs 26.08 (±11.33) | **0.001** | 26.73 (±12.16) vs 26.99 (±12.07) | 0.968 |
| PLT/WBC >25 | 1.64 (1.20 - 2.24) | **0.002** | 0.82 (0.50 - 1.38) | 0.519 |
| WBC/MPV | 0.94 (±0.45) vs 1.11 (±0.45) | **<0.0001** | 1.05 (±0.50) vs 1.07 (±0.45) | 0.527 |
| WBC/MPV <0.9 | 2.69 (1.87 - 3.88) | **<0.0001** | 1.12 (0.63 - 2.00) | 0.768 |

Abbreviations: OR-odds ratio, CI- confidence interval, SD – standard deviation, BMI-Body mass index, GTR-Gross total resection, GFAP- Glial fibrillary acidic protein immunohistochemistry staining percentage, MGMT- O[6]-methylguanine-DNA methyltransferase promoter methylation, IDH1-Isocitrate dehydrogenase 1 mutation, KPS – Karnofsky Performance Scale, WBC-White blood cells, MCV- mean corpuscular volume, MCH- Mean Corpuscular Hemoglobin, MCHC- mean corpuscular hemoglobin concentration, PLT- blood platelets, MPV- Mean platelet volume, PT- Prothrombin time, aPTT- activated partial thromboplastin time, ASAT- Aspratate transaminase, ALT- Alanine transaminase, ALP- Alkaline phosphatase, GGT- Gamma-glutamyltransferase, LDH- Lactate dehydrogenase, CRP- C reactive protein, GFR- Glomerular filtration rate, APRI- Aspartat amino transferase to platelet ratio

**Table S2:** Multivariate analysis consisting of the laboratory markers associated with the SAO and EPS

| Parameter | aOR | 95% CI | | p-value |
| --- | --- | --- | --- | --- |
|  |  | Lower | Upper |  |
| Predictors of SAO | | | | |
| WBC >11 /nl | 0.610 | 0.281 | 1.323 | 0.211 |
| PLT >400 /nl | 0.469 | 0.131 | 1.681 | 0.245 |
| Sodium <140 mmol/l | 1.410 | 0.868 | 2.290 | 0.165 |
| Chloride >102 U/l | 1.978 | 1.209 | 3.236 | 0.007 |
| Calcium >2.35 mmol/l | 1.670 | 1.060 | 2.632 | 0.027 |
| Urea >20 mg/dl | 0.749 | 0.476 | 1.180 | 0.212 |
| Total protein <7.0 g/dl | 0.726 | 0.460 | 1.145 | 0.168 |
| PLT/WBC >25 | 0.695 | 0.398 | 1.213 | 0.200 |
| WBC/MPV <0.9 | 1.876 | 0.926 | 3.798 | 0.081 |
| Predictors of EPS | | | | |
| Hemoglobin <12 g/dl | 3.023 | 1.023 | 8.935 | 0.045 |
| Calcium >2.35 mmol/l | 0.723 | 0.210 | 2.488 | 0.607 |
| GGT >55 U/l | 2.566 | 1.053 | 6.254 | 0.038 |
| Total protein <7.0 g/dl | 1.427 | 0.521 | 3.909 | 0.490 |
| CRP >1.0 mg/dl | 3.316 | 1.338 | 8.220 | 0.010 |

**Abbreviations**: aOR- adjusted odds ratio, CI- Confidence interval, WBC- White blood cells, PLT- blood platelets, GGT- Gamma-glutamyltransferase, CRP- C-reactive protein

**Table S3:** Multivariate analysis of EPS predictors replacing anemia marker (low hemoglobin through red blood cells count)

| Parameter | aOR | 95% CI | | p-value |
| --- | --- | --- | --- | --- |
|  |  | Lower | Upper |  |
| Location: frontal/temporal lobe | 2.296 | 1.182 | 4.461 | **0.014** |
| Comorbidity: Arterial hypertension | 1.564 | 0.882 | 2.776 | 0.126 |
| Comorbidity: Second malignancy | 1.782 | 0.941 | 3.376 | 0.076 |
| Blood test: red blood cells count <4 /nl | 2.649 | 1.041 | 6.742 | **0.041** |
| Blood test: GGT >55 U/l | 1.876 | 1.043 | 3.374 | **0.036** |
| Blood test: CRP >1.0 mg/dl | 2.437 | 1.088 | 5.461 | **0.033** |

**Abbreviations**: aOR- adjusted odds ratio, CI- Confidence interval, GGT- Gamma-glutamyltransferase, CRP- C-reactive protein

**Table S4:** Cox multivariate analysis of OS predictors using different perioperative seizures endpoints (SAO or EPS) and other common outcome confounders

| Parameter | aOR | 95% CI | | p-value | aOR | 95% CI | | p-value |
| --- | --- | --- | --- | --- | --- | --- | --- | --- |
|  |  | Lower | Upper |  |  | Lower | Upper |  |
| * SAO | | | | | * EPS | | | |
| Age. years | 1.024 | 1.014 | 1.035 | **<0.0001** | 1.026 | 1.015 | 1.036 | **<0.0001** |
| Perioperative seizures* | 0.824 | 0.693 | 0.981 | **0.030** | 1.413 | 1.090 | 1.830 | **0.009** |
| KPS % | 0.987 | 0.979 | 0.994 | **0.001** | 0.986 | 0.978 | 0.993 | **<0.0001** |
| EOR | 0.441 | 0.370 | 0.525 | **<0.0001** | 0.432 | 0.364 | 0.514 | **<0.0001** |
| MGMT methylation | 0.700 | 0.573 | 0.855 | **0.001** | 0.693 | 0.565 | 0.850 | **0.001** |
| IDH1 mutation | 0.740 | 0.209 | 2.628 | 0.568 | 0.726 | 0.207 | 2.546 | 0.540 |
| Chemoradiation | 0.414 | 0.332 | 0.516 | **<0.0001** | 0.421 | 0.337 | 0.525 | **<0.0001** |

Abbreviations: aOR- Adjusted odds ratio, CI- Confidence interval, KPS- Karnofsky performance scale, EOR- Extent of resection, MGMT- O[6]-methylguanine-DNA methyltransferase promoter methylation, IDH1-Isocitrate dehydrogenase 1 mutation
